# The CSF‐Inventory: A Self‐Reported Assessment Tool for Spinal Cerebrospinal Fluid (CSF) Leak Symptoms and Functional Impact

**DOI:** 10.1111/ene.70809

**Published:** 2026-11-05

**Authors:** Katharina Wolf, Amir El Rahal, Christian Fung, Myriam Peters, Niklas Lützen, Eike I. Piechowiak, Tomas Dobrocky, Manou Overstijns, Matthias Otte, Christian Moeller, Charlotte Zander, Vanessa Vieira da Silva, Horst Urbach, Jürgen Beck, Florian Volz

**Affiliations:** ^1^ Department of Neurosurgery Medical Center, University of Freiburg Freiburg Germany; ^2^ Lindenhofspital Bern Switzerland; ^3^ Department of Neuroradiology Medical Center, University of Freiburg Freiburg Germany; ^4^ Department of Neuroradiology Inselspital, University of Bern Bern Switzerland; ^5^ Neurocenter‐IT‐Group, Department of Neurosurgery Medical Center, University of Freiburg Freiburg Germany; ^6^ Department of Radiology Medical Center, University of Freiburg Freiburg Germany

**Keywords:** headache, patient‐reported outcome measures, post‐dural puncture headache (PDPH), social impact, spinal CSF leak, spontaneous intracranial hypotension (SIH)

## Abstract

**Background:**

Patients with spontaneous intracranial hypotension (SIH) or post‐dural puncture headaches (PDPH) frequently experience a variety of symptoms such as orthostatic headaches, cognitive decline, vestibulocochlear and visual disturbances, that may lead to physical, mental, and social burden. Despite increasing awareness, standardized tools for symptom assessment are lacking. This study aims to provide a framework for reporting the heterogeneous symptoms and to quantify the burden associated with spinal CSF leaks.

**Methods:**

The conceptual development and structure of the CSF‐Inventory, a patient‐reported outcome measure (PROM) were designed via a modified Delphi consensus through a bicentric panel including experts from neurology, neurosurgery, neuroradiology, and dedicated nursing, all with extensive experience in spinal CSF leaks. Patient feedback guided the item generation and revision. Short‐interval response agreement was explored in 20 patients via intraclass correlation coefficient (ICC) and Cohen's kappa as appropriate.

**Results:**

The CSF‐inventory instrument comprises 18 items clustered into three domains: (1) functional limitations, (2) symptom profile, and (3) symptom severity. The resulting tool captures key features such as working capacity, daily upright tolerance, headache frequency and severity, as well as the presence and severity of other specified symptoms. Repeated testing yielded almost identical results (ICC/Kappa > 0.98), underscoring basic clarity and feasibility.

**Conclusion:**

The CSF‐Inventory provides a standardized framework for assessing CSF leak‐related symptoms by bridging expert and patient experiences. As such, it may provide metrics for longitudinal monitoring and the definition of patient‐centered study endpoints.

## Graphical abstract

Graphical Abstract—Summary—CSF Inventory: Spontaneous intracranial hypotension (SIH) is more than a headache, affecting multiple symptom domains, daily functioning, and quality of life. Developed through expert consensus and patient input, the CSF‐Inventory provides a standardized, multidimensional patient‐reported assessment of symptoms, upright time, and work capacity. It aims to capture the full burden of SIH beyond headache and establish a foundation for more comprehensive assessment of disease severity and treatment outcomes.

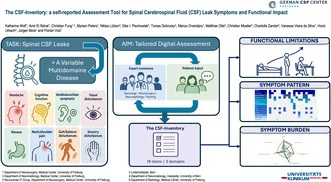

## 1 Introduction

Spinal cerebrospinal fluid (CSF) leaks allow CSF to escape from the intrathecal space. These defects can occur spontaneously in spontaneous intracranial hypotension (SIH), or they derive from iatrogenic interventions causing the syndrome of a post‐dural puncture headache (PDPH). Over the past decade, there has been exponential progress in the recognition, diagnostic work‐up, and treatment of SIH, a historically underrated and underrecognized disease [1, 2, 3]. It is well known that the clinical presentation of spinal CSF leaks can involve a multitude of functionally impairing domains reaching from the most recognized feature of orthostatic headaches to severe cognitive impairments [1, 4, 5]. Clinical recognition therefore may remain challenging for physicians, especially as a relevant proportion of patients may present without orthostatic headaches and with a change of orthostatic features over time [5, 6, 7]. Misdiagnoses range from migraine to psychosomatic disorders [8, 9]. Chronic courses of PDPH (cPDPH) have been delineated with clinical similarities highlighting an often underestimated and disabling condition following an intervention, underscoring the need for further in‐depth research [10, 11, 12, 13]. Patients living with these diseases report significant limitations in daily life, social functioning, and mental distress [14, 15, 16]. Importantly, the population affected by SIH or cPDPH is predominantly in their working years [1], and although the condition is debilitating, it is treatable and curable in a majority of cases [14, 15, 16, 17].

A restricted focus on orthostatic headache may overlook patients whose chief complaints manifest through other disabling symptoms. At present, symptom information is typically gathered through patient histories, headache‐dominated tools, and non‐disease‐specific comparators, generalizable patient‐reported outcome measure (PROM) tools [14, 15, 18, 19].

There is a need for a comprehensive yet practical framework to uniformly assess the patient's symptom frequency and burden in spinal CSF leaks across all patients and different stages.

The objective of this study is to propose the CSF‐Inventory as a dedicated patient‐reported outcome measure (PROM) for spinal CSF leak syndromes.

## 2 Methods

The development of the CSF‐Inventory followed a modified Delphi consensus approach informed by a multidisciplinary group of clinicians with extensive experience in CSF centers, specialized nursing staff, and patient feedback. The process was conducted at two tertiary referral centers based in Germany and Switzerland with cumulative experience in evaluating and treating several 100 patients with confirmed spinal CSF leaks (post‐dural puncture headache [PDPH] and spontaneous intracranial hypotension [SIH]) between October 2023 and June 2024.

Confirmation of such leaks was based on unequivocal radiological evidence of SIH and classification of the leak depicted by adequate imaging techniques as published before [12, 13, 20, 21, 22, 23, 24, 25], or via imaging and/or intraoperative confirmation in PDPH and cPDPH.

The design of the CSF‐Inventory items was guided by a broad conceptualization of the clinical spectrum of SIH and PDPH in both acute and chronic presentations [1, 6, 9, 18, 26, 27]: we presumed three premises: the patient's presentation is shaped not only by physiological factors (i.e., the direct symptoms and physical effects of the CSF leak), but also by psychosocial factors and socioeconomic factors (such as the ability to work).

The primary item pool was generated by a neurological and neurosurgical core team based on expert opinion, patient consultation, and experience from two preceding, self‐created questionnaire prototypes.

- Pre‐Version 1: An initial prototype questionnaire was self‐created in 2019. This first version was an extended survey that inquired about a range of specific, prior described typical symptoms reported in the context of spinal CSF leaks, such as orthostatic headaches, neck pain, auditory and vestibular symptoms (as yes/no items) [1, 6].
- Pre‐Version 2: A substantially condensed second version was introduced in 2021 with mainly open‐ended self‐report fields that allowed patients to describe symptoms in their own words. In addition, validated patient‐reported outcome measures (PROMs) were used as adjuncts to quantify headache burden and generic health‐related quality of life, including the 6‐Item Headache Impact Test (HIT‐6) [28] and EuroQol (EQ‐5D‐5L) [29]. The inclusion of these validated comparator measures was intended to enable comparisons between CSF leak patients and other populations [30].

In constructing the CSF‐Inventory item pool, several items were added or refined to better quantify aspects of the condition that emerged as important to experts and patients alike: e.g., an item to record the maximum duration a patient can remain upright continuously, which is a distinctive functional limitation in CSF leak syndrome. Furthermore, cognitive dysfunction was addressed [31]. Besides typical headache outcome metrics, work capacity was incorporated as a key item to gauge the socioeconomic impact [19, 27].

The expert panel (4 neurosurgeons, 1 neurologist, 4 neuroradiologists, 1 neuroscientist, 1 nurse specialist; *n* = 11) participated in three iterative consensus rounds (Figure 1). Items achieving ≥ 80% agreement were retained, while others were revised or removed in subsequent rounds. In each round, panel members assessed item relevance and clarity and provided feedback. The initial pool comprised 22 items. The content of four items was either added to others or combined into one item; one item was not retained. Mean agreement for the final 18 items was 95%, with all retained items meeting the predefined ≥ 80% threshold. To incorporate the patient perspective, informal individual feedback discussions were conducted with 10 affected patients under care prior to the first and before the final expert consensus round. Patients were asked whether the questions were understandable, whether their symptoms and burden were adequately represented, and whether important concerns were missing. The direct feedback loop from patients led to several refinements: for instance, we added free‐text fields for certain sections so that patients could report any symptom or concern not explicitly covered by the checklist items.

**FIGURE 1:**
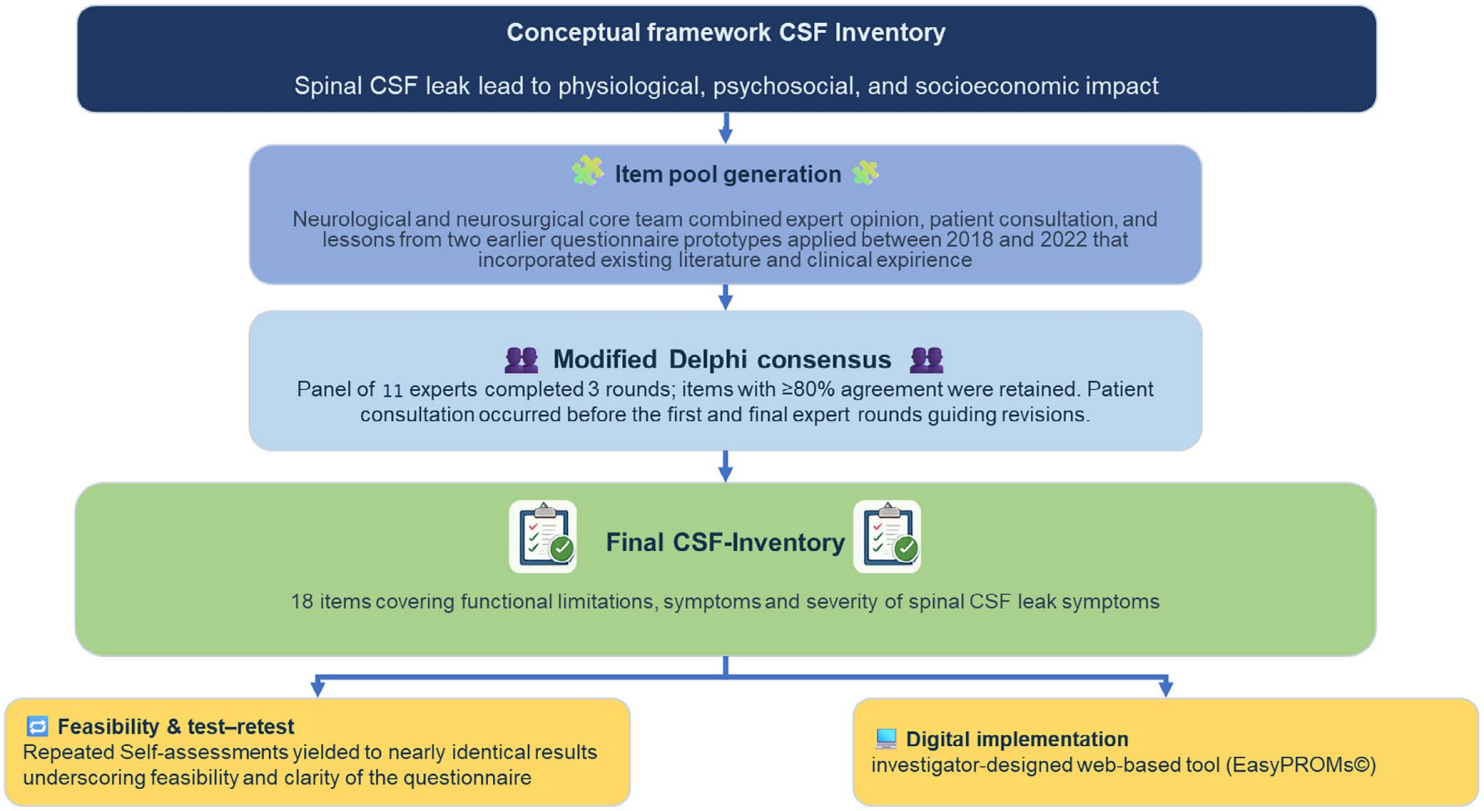
Development of the CSF‐Inventory through a modified Delphi consensus approach across experts with extensive experience in spinal CSF leaks. Item generation was informed by expert opinion, longitudinal clinical observation, and patient consultation and feedback.

Finally, the questionnaire was incorporated into an internally investigator‐designed web‐based digital tool (easyPROMs).

Feasibility and short‐interval response agreement of the questionnaire were assessed using a test–retest design. Twenty patients were asked to complete the questionnaire twice within an interval of no longer than 14 days to evaluate item comprehensibility and response stability. Short‐interval response agreement of the quantitative items was rated using the intraclass correlation coefficient (ICC) based on a two‐way mixed‐effects model assessing absolute agreement. ICC values greater than 0.90 were reported as indicating high agreement. For categorical variables, agreement was assessed using Cohen's kappa statistics. Values above 0.80 for kappa were reported as indicating high agreement.

### 2.1 Ethical Considerations

The development of the CSF‐Inventory between 2019 and 2024 was conducted as part of a quality improvement initiative with institutional approval (Ethic #22‐1512_1‐S1‐retro). As the instrument is further used within the registry for spinal CSF leaks (SEAL #NCT06711731), formal research ethics approval was prospectively secured (Ethic #24–1357‐S1).

## 3 Results

The final CSF‐Inventory consists of 18 items organized into three major domains, reflecting the multidimensional impact of spinal CSF leaks.

All items in the CSF‐Inventory are patient‐reported and were designed for self‐administration. The full structured version of the CSF‐Inventory, including all item wording and response options as described below in summary form, is provided as Data S1.

### 3.1 Domain 1: Functional Limitations (Quantitative)

#### 3.1.1 Focus

Occupational and daily functioning, upright posture tolerance, and fatigue (items 1, 10, 17, and 18). This domain encompasses the patient‐reported limitations in work capacity, endurance in upright posture measured in continuous upright time, and overall energy levels in daily life (fatigue). It captures how the CSF leak syndrome affects the ability to perform normal roles and routines.

### 3.2 Domain 2: Symptom Profile (Qualitative)

#### 3.2.1 Focus

Presence of specific symptoms, most prominent complaints, and headache comparators (items 2, 3, 4 & 7, 8, 9, Figure 2). This domain assesses which symptoms the patient is experiencing, including checklists on current symptoms assessed in yes/no, the most stressful symptom at the time, positional and diurnal aspects of the symptoms, and headache location. Furthermore, some additional quantitative metrics such as headache days per month, days with medication intake facilitate comparison to other headache conditions and objective tracking of the course of the disease. It aims to capture the wide variety of the symptomatology and identify which symptom is the most burdensome for the patient. The possibility to report other symptoms not specifically asked for is provided.

**FIGURE 2:**
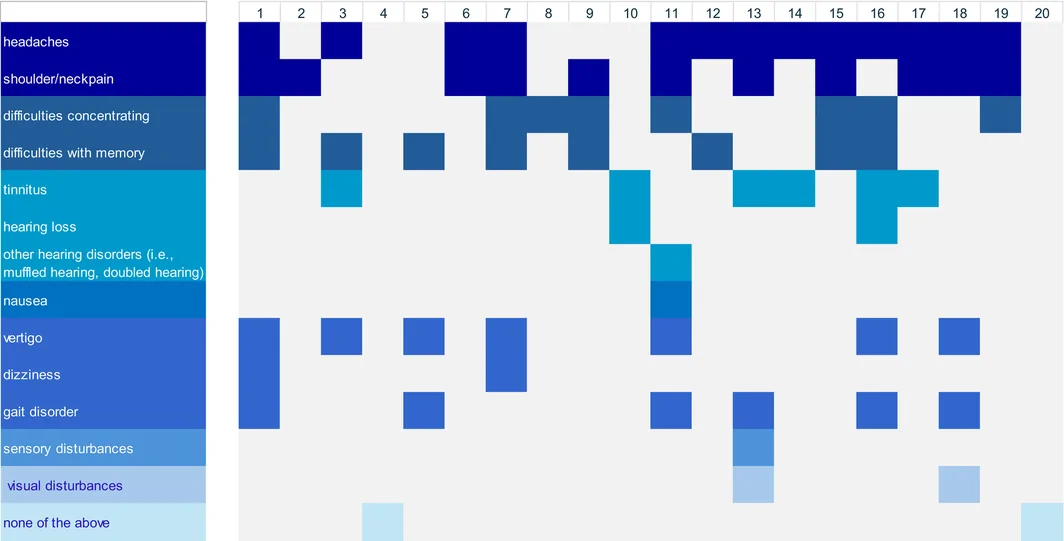
The breadth of symptoms of patients with spinal CSF leaks (patients 1–20); colored squares indicate the presence of a symptom. This type of analysis will provide data for specific analysis of symptom patterns potentially delineating the spectrum of the diseases.

### 3.3 Domain 3: Symptom Severity (Quantitative)

#### 3.3.1 Focus

Quantitative measures of symptom severity (items 5, 11–16, Figure 3). This domain captures how intense the symptoms are and how much they are affecting the patient in measurable/comparable terms. Quantitative symptom burden measures are bundled into functional domains (e.g., dizziness, vertigo, and gait instability/ataxia as one) that are commonly described interchangeably by patients. All items ask for the average severity of symptoms over the past 4 weeks on a 0 to 10 scale, with 10 being the highest intensity.

**FIGURE 3:**
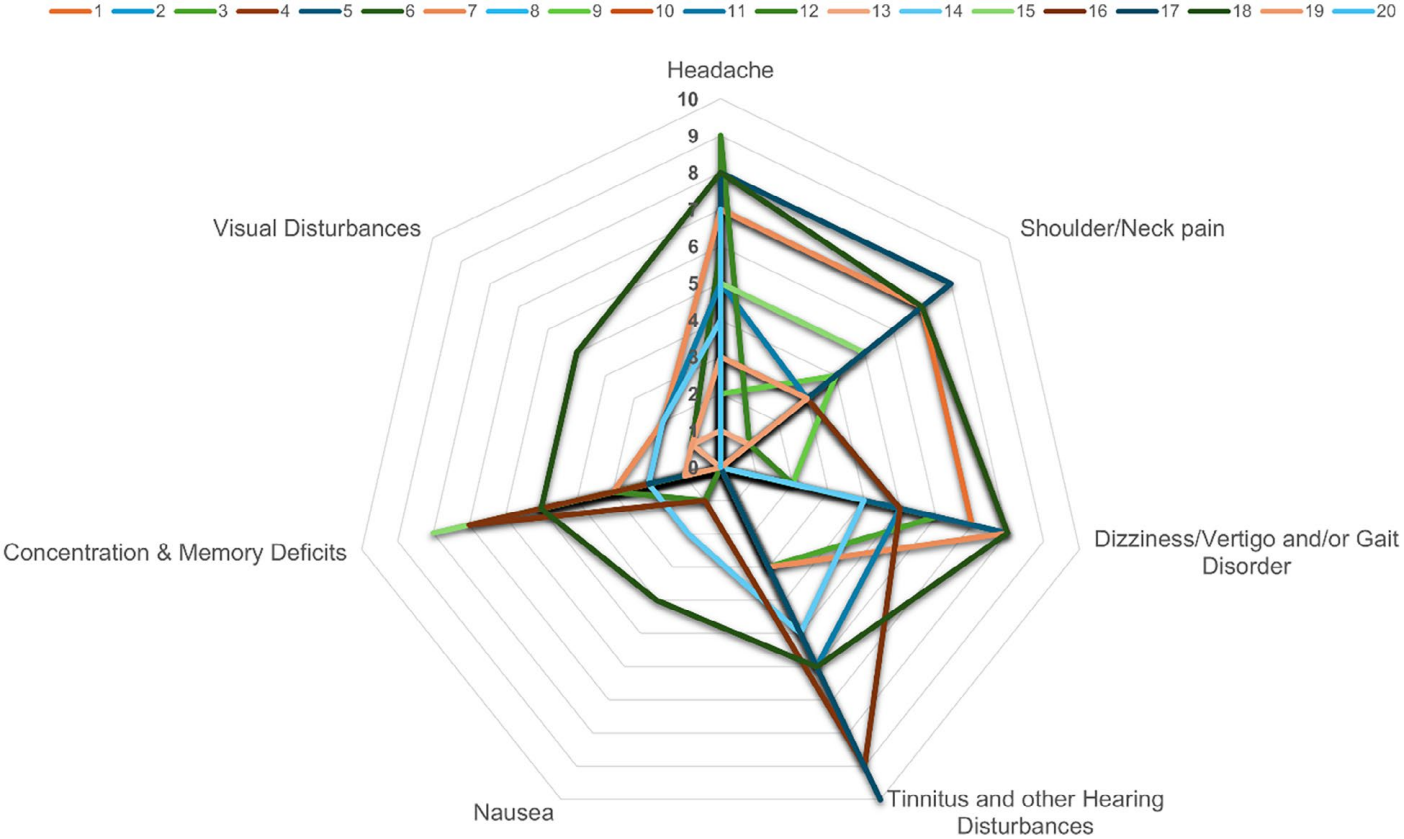
Severity and variability of patient‐reported syndromes rated between 0 = “not at all” to a maximum of 10 = “extremely” severe over the course of the past 4 weeks. Each patient (*n* = 20) is represented by one colored line displaying the individual burden. The high variability between patients with proven and ongoing leaks and their respective variability of symptom burden can be appreciated, which underscores the need for a more detailed, yet feasible assessment tool.

### 3.4 Scoring and Interpretation

At this phase, the CSF‐Inventory does not produce one single sum score, yet provides several quantitative metrics suitable for comparison (e.g., average of numeric severity scales, headache severity and headache days, social impact, etc.). Categorical responses (such as those in items 1–4, 6, 8, 9, 17, and 18) are intended for descriptive use or to identify particular symptom patterns, such as between leak types and over the duration of the disease, or risk of comorbidity such as medication overuse headache. Thus, the current output is a structured profile rather than one number or category. Numeric measures can be compared item by item within patients over time or between treatment groups, while categorical variables can be compared as proportions or used for pattern identification in larger datasets.

### 3.5 Short‐Interval Response Agreement

Turnaround time for the re‐test assessment was a median of 2 days (range 0.5–14 days) resulting in close to identical responses that took no longer than 15 min per test (ICCs > 0.98, Cohen's kappa > 0.9, Table S1). Given the brief interval, including same‐day reassessment, these findings may be influenced by recall and should not be interpreted as establishing test–retest reliability or validation.

### 3.6 Comparator Instruments: (Quantitative)

The CSF‐Inventory is intended to complement established measures. We propose supplementing the CSF‐Inventory with comprehensive comparator PROM metrics to capture the details of leak symptoms while allowing comparison across diseases. This includes combination with generic tools like the EQ‐5D‐5L to measure health‐related quality of life [29], as well as headache‐specific (originally migraine) measures such as the HIT‐6 or similar tools. In our current clinical implementation, the CSF‐Inventory is administered alongside a small battery of established questionnaires for comprehensive assessment of comorbidity (self‐reported comorbidity questionnaire—SCQ [32]), the EQ‐5D‐5L, and the Clinical Global Impression scale after treatment (CGI) [33]. These external measures are not part of the CSF‐Inventory per se, but their inclusion is considered useful for overall quality of life metrics, and to rate outcome after intervention, adding about five to 10 min to the completion of the CSF‐Inventory.

## 4 Discussion

The CSF‐Inventory represents a structured tool for the assessment of spinal CSF leak symptoms. Its content expands to include cognitive difficulties, vestibulocochlear, sensory and visual problems, orthostatic tolerance, and social and functional impact in addition to headache metrics. With this tool, the clinical CSF fingerprint evolving over the course of the diseases might be further delineated, that is frequently observed in clinical practice and that might further frame the described changes from onset to chronicity [7]. The heterogeneity across a small patient cohort with proven and ongoing leaks depicted and illustrated in Figures 2 and 3 underlines this rationale. Individual numeric ratings can be followed longitudinally, while larger datasets may identify recurring combinations of symptom patterns that are associated with disease duration, leak type, or time course of often delayed treatment response. Whether such patterns define clinically meaningful subgroups or predict outcome requires prospective validation.

Another potentially beneficial aspect of the item pool is the potential to uncover the variety of clinical presentations and broaden the conventional diagnostic perspective as further testing of the CSF‐Inventory evolves. Typically, SIH is traditionally defined by the presence of clinical criteria as a prerequisite, most prominently, the orthostatic headache as per ICHD‐3 criteria described [4] that classifies headache conditions yet not the disease with its multitude of impairing symptoms itself. The instrument and its further developments therefore might help to capture the “real‐world” phenotype of spinal CSF leaks. Additionally, as data accumulate, we may be able to explore potential differences between subgroups of patients, and variability by leak type, sex, and age.

The short‐term response stability underscores that patients do understand the questionnaire and can answer it clearly. Yet, further validations are needed across centers and languages, including larger sets and within‐item validation. Observations and item validations across longer time spans may likely encounter a higher variability of symptoms, which lies in the nature of the disease and its dynamic clinical presentation [7]. Importantly, the median retest interval of 2 days, same‐day reassessment in some participants, and the small sample frame create a substantial risk of recall bias. Conversely, longer intervals in SIH may capture true day‐to‐day symptom fluctuation. The present findings therefore demonstrate only short‐interval response agreement and item comprehensibility, not established test–retest reliability or validation. Future studies should use larger cohorts, prespecified longer retest intervals, and concurrent recording of treatment and clinical change.

Notably, the CSF‐Inventory is intended as a symptom and impact assessment tool for patients with CSF leaks, rather than as a diagnostic checklist or discriminator tool. By characterizing patients' social, symptomatic, and functional burden through this instrument, we seek to capture the multitude of clinical presentations of patients with CSF leaks in a standardized way, potentially contributing to improved diagnostics in the future. The instrument is intended to complement, not replace, established headache and generic quality‐of‐life measures.

## 5 Conclusion

The CSF‐Inventory is a novel patient‐reported outcome instrument designed for spinal CSF leak syndromes, including spontaneous intracranial hypotension and post‐dural puncture headache. It bridges a gap in the field by systematically capturing the variety of symptoms and the associated burden in this patient population. Following further validation, this instrument could be used to track patient outcomes over time, evaluate responses to interventions, and facilitate multicenter research by providing a common language of symptom measurement.

## Author Contributions

**Katharina Wolf:** conceptualization, investigation, funding acquisition, writing – original draft, methodology, validation, software, project administration, data curation, visualization, formal analysis. **Amir El Rahal:** conceptualization, investigation, writing – review and editing, project administration. **Christian Fung:** conceptualization, investigation, writing – review and editing. **Myriam Peters:** conceptualization, investigation, writing – review and editing, methodology, data curation, writing – original draft. **Niklas Lützen:** conceptualization, investigation, writing – review and editing. **Eike I. Piechowiak:** conceptualization, investigation, writing – review and editing, methodology. **Tomas Dobrocky:** conceptualization, investigation, methodology, writing – review and editing. **Manou Overstijns:** conceptualization, investigation, writing – review and editing. **Matthias Otte:** conceptualization, investigation, software, writing – review and editing, project administration. **Christian Moeller:** conceptualization, investigation, writing – review and editing, software, project administration. **Charlotte Zander:** conceptualization, investigation, writing – review and editing. **Vanessa Vieira da Silva:** conceptualization, investigation, project administration. **Horst Urbach:** conceptualization, investigation, writing – review and editing, resources, methodology. **Jürgen Beck:** conceptualization, investigation, writing – review and editing, methodology, resources, supervision. **Florian Volz:** conceptualization, writing – review and editing, methodology, supervision, resources, investigation.

## Funding

This work has not received external funding.

## Disclosure

Amir El Rahal received a scholarship grant from the Michel et André Bouriez Foundation. Katharina Wolf is a fellow of the Margarete‐von‐Wrangell Program, Ministry of Research, Science, and Art, Baden‐Württemberg, Germany.

## Conflicts of Interest

The authors declare no conflicts of interest.

## Supporting information


**Data S1:** The CSF inventory—CSF Center Freiburg.

**Table S1:** Table displaying test–retest‐results of the CSF‐Inventory in 20 patients with confirmed spinal CSF leaks.

## Data Availability

The data that supports the findings of this study are available in the Supporting Information of this article.
