# Supplementary material for: The CSF‐Inventory: A Self‐Reported Assessment Tool for Spinal Cerebrospinal Fluid (CSF) Leak Symptoms and Functional Impact
Source: Eur J Neurol. 2026 Nov 5;33(11):e70809. doi: 10.1111/ene.70809 (PMC13740670; doi:10.1111/ene.70809)
Supplement: Supplementary file 1 [file ENE-33-e70809-s001.docx]

THE CSF INVENTORY – CSF Center Freiburg

**Instructions for Use**

The CSF-Inventory is a patient-reported screening questionnaire designed to assess symptoms, functional capacity, and social impact in individuals with suspected or confirmed cerebrospinal fluid (CSF) disorders.
Patients are asked to answer each question according to their current condition and recent experience (past four weeks).

Response formats include multiple-choice, Likert-type, and numeric rating scales.

The rating for statistical assessments is here given in () which are not displayed to the participant answering the questionnaire.

**Scoring and Interpretation**

Items with numeric scales assessing quantitative symptom burden on a 0–10 scale can be averaged for a global symptom severity score (items 5 & 11-16).

Rankings are reported descriptively, with the option to include them in a proportional sum score.
Categorical responses are analyzed descriptively or used for pattern identification.

Overall: Higher numeric ratings indicate higher symptom burden or functional impairment.

**Terms of use:**

The CSF-Inventory may be used for research and clinical purposes with appropriate citation to the original publication and reference to the Freiburg CSF Center

Permission to use the CSF-Inventory for industrial purposes or clinical trials must be requested via: nez.sih@uniklinik-freiburg.de

CSF Inventory Questionnaire***:**

** The variables used for statistical assessment are provided in parentheses and are not displayed to participants*

Dear Participant,

Thank you for taking the time to answer the following questions with regard to your illness that help to capture your current disease burden.

The questions refer to your current state and severity of your symptoms during the past four weeks as stated in the introduction to each question. Your symptoms might have changed over time – your medical team is aware of this. There is no need to take prior symptoms into account.

**Items**

**1)** Current professional working capacity (single choice, ranking)
☐ I can work at full capacity (0)
☐ I work at full capacity, but with adaptions(e.g., home office, lying down at the office, taking breaks) (1)
☐ I have reduced working capacity due to current symptoms to 50–80% (2)
☐ I have reduced working capacity due to current symptoms to 20–50% (3)
☐ I am on sick leave (4)
☐ I do not like to share this information (5)

**2)** My current symptoms are (multiple choice, qualitative)
☐ Headaches (1)
☐ Shoulder/neck pain (2)
☐ Difficulties concentrating (3)
☐ Difficulties with memory (4)
☐ Tinnitus (5)
☐ Hearing loss (6)
☐ Other hearing disorders (e.g., muffled hearing, double hearing) (7)
☐ Nausea (8)
☐ Vertigo (9)
☐ Dizziness (10)
☐ Gait disorder (11)
☐ Sensory disturbances (12)
☐ Visual disturbances (13)
☐ None of the above (14)

☐ Others – please describe (free text option)

**3)** My most stressful symptom is (single choice, qualitative)
☐ Headaches (1)
☐ Shoulder/neck pain (2)
☐ Difficulties concentrating (3)
☐ Difficulties with memory (4)
☐ Tinnitus (5)
☐ Hearing loss (6)
☐ Other hearing disorders (e.g., muffled hearing, double hearing) (7)
☐ Nausea (8)
☐ Vertigo (9)
☐ Dizziness (10)
☐ Gait disorder (11)
☐ Sensory disturbances (12)
☐ Visual disturbances (13)
☐ None of the above (14)

☐ Others – as stated above

**4)** Over the past four weeks, my symptoms - (multiple choice, qualitative)
☐ Improve by at least 50% when lying flat (1)
☐ Improve by at least 50% when standing upright (2)
☐ Worsen when moving (3)
☐ Improve when moving (4)
☐ Are worst in the morning (5)
☐ Are worst in the evening (6)
☐ Are similar throughout the entire day (7)
☐ Differ every day (8)

**5)** Within the past four weeks, my headaches are on average [0 – not at all to 10 = extremely] painful. (single choice, quantitative)

(quantitative - 0-10)

**6)** Within the past four weeks, my headaches are most severe (multiple choice, qualitative)
☐ At the back of my head (1)
☐ At the front / behind the eyes (2)
☐ At the sides (3)
☐ In the entire head (4)
☐ I do not have headaches (5)

**7)** Days with headache within the last month [Numeric Scale: 0–30] (single choice, quantitative)

(qualitative - 0-30)

**8)** Days with pain medication intake within the last month (single choice, categorial)
☐ 0–5 (0)
☐ 6–10 (1)
☐ 11–14 (2)
☐ 15–20 (3)
☐ 21–25 (4)
☐ 26–31 (5)

**9)** I take the following pain treatments
☐ Over-the-counter pain medication
☐ Prescribed pain medication (e.g metamizole / Novaminsulfon)
☐ Opioids
☐ Oral Pain-modulating therapies (prevention - e.g., amitriptyline, pregabaline, gabapentin)
☐ Triptans
☐ Botox injections
☐ Occipitalis injections
☐ Others
☐ None

**10)** At daytime, the maximum duration I can remain continuously upright is [Numeric Scale: 0–16 hours] (single choice, quantitative)

(quantitative – 0-16)

**11)** During the past four weeks, the dizziness/vertigo and/or gait disorder are on average [0= not, 10=extremely] stressful. (single choice, quantitative)

(quantitative - 0-10)

**12)** During the past four weeks, my shoulder/neck pain is on average [0= not, 10=extremely] severe. (single choice, quantitative)

(quantitative - 0-10)

**13)** During the past four weeks, the nausea is on [0= not, 10=extremely] severe. (single choice, quantitative)

(quantitative - 0-10)

**14)** During the past four weeks, my hearing disturbances/tinnitus are on average [0= not, 10=extremely] stressful. (single choice, quantitative)

(quantitative - 0-10)

**15)** During the past four weeks, my deficits in concentration and memory are on average [0= not, 10=extremely] severe. (single choice, quantitative)

(quantitative - 0-10)

**16)** During the past four weeks, my visual disturbances are on average [0= not, 10=extremely] stressful. (single choice, quantitative)

(quantitative - 0-10)

**17)** I am very exhausted (single choice, ranking)
☐ Never (0)
☐ Rarely (1)
☐ Sometimes (2)
☐ Very often (3)
☐ Always (4)

**18)** If I do something, I can generally focus and concentrate. (single choice, ranking)
☐ Never (4)
☐ Rarely (3)
☐ Sometimes (2)
☐ Very often (1)
☐ Always (0)
